# Frizzled-8 receptor is activated by the Wnt-2 ligand in non-small cell lung cancer

**DOI:** 10.1186/1471-2407-13-316

**Published:** 2013-07-01

**Authors:** Dawn T Bravo, Yi-Lin Yang, Kristopher Kuchenbecker, Ming-Szu Hung, Zhidong Xu, David M Jablons, Liang You

**Affiliations:** 1 Department of Surgery, Helen Diller Family Comprehensive Cancer Center, University of California, 2340 Sutter Street, N-221, San Francisco, CA 94115, USA

**Keywords:** Frizzled-8, Wnt-2, dnhWnt-2 Construct, Lung Cancer, Wnt Signaling

## Abstract

**Background:**

Wnt-2 plays an oncogenic role in cancer, but which Frizzled receptor(s) mediates the Wnt-2 signaling pathway in lung cancer remains unclear. We sought to (1) identify and evaluate the activation of Wnt-2 signaling through Frizzled-8 in non-small cell lung cancer, and (2) test whether a novel expression construct dominant negative Wnt-2 (dnhWnt-2) reduces tumor growth in a colony formation assay and in a xenograft mouse model.

**Methods:**

Semi-quantitative RT-PCR was used to identify the expression of Wnt-2 and Frizzled-8 in 50 lung cancer tissues from patients. The TCF reporter assay (TOP/FOP) was used to detect the activation of the Wnt canonical pathway *in vitro*. A novel dnhWnt-2 construct was designed and used to inhibit activation of Wnt-2 signaling through Frizzled-8 in 293T, 293, A549 and A427 cells and in a xenograft mouse model. Statistical comparisons were made using Student’s *t*-test.

**Results:**

Among the 50 lung cancer samples, we identified a 91% correlation between the transcriptional increase of Wnt-2 and Frizzled-8 (*p*<0.05). The Wnt canonical pathway was activated when both Wnt-2 and Frizzled-8 were co-expressed in 293T, 293, A549 and A427 cells. The dnhWnt-2 construct we used inhibited the activation of Wnt-2 signaling in 293T, 293, A549 and A427 cells, and reduced the colony formation of NSCLC cells when β-catenin was present (*p*<0.05). Inhibition of Wnt-2 activation by the dnhWnt-2 construct further reduced the size and mass of tumors in the xenograft mouse model (*p*<0.05). The inhibition also decreased the expression of target genes of Wnt signaling in these tumors.

**Conclusions:**

We demonstrated an activation of Wnt-2 signaling via the Frizzled-8 receptor in NSCLC cells. A novel dnhWnt-2 construct significantly inhibits Wnt-2 signaling, reduces colony formation of NSCLC cells *in vitro* and tumor growth in a xenograft mouse model. The dnhWnt-2 construct may provide a new therapeutic avenue for targeting the Wnt pathway in lung cancer.

## Background

Lung cancer is the most commonly diagnosed malignancy worldwide and is responsible for over one million deaths each year [1,2]. Current treatment strategies include surgical resection, chemotherapy, radiation therapy, targeted therapy, or a combination of treatments, depending on disease type and stage [1,2]. Despite advances in multimodality treatments, lung cancer remains highly lethal, with a 5-year survival rate of less than 15% [2]. New treatment strategies are urgently needed.

Wnt signaling elicits numerous cellular responses including self-renewals of stem cells [3]. Currently, 10 Frizzled proteins have been identified in mammals as the receptors for Wnt proteins. Transduction of Wnt signaling begins when Wnt ligands bind to the cysteine-rich Wnt binding domain (CRD) of Frizzled receptors at the cell membrane and initiate either the ‘canonical’ or ‘non-canonical’ pathways [4]. The canonical Wnt signaling pathway regulates the stability of β-catenin [5]. When Wnt is not activated, β-catenin is phosphorylated by the destruction complex and degraded by ubiquitination [6,7]. When binding to Frizzled receptors and low-density lipoprotein co-receptors 5 and 6 (LRP5/6) on cell membrane [8,9], Wnt signaling is activated and Dishevelled (DVL) recruits the destruction complex to the plasma membrane [10], resulting in β-catenin stabilization and subsequent accumulation in the cytoplasm [5]. Stabilized β-catenin then enters the cell nucleus and associates with lymphoid enhancer-binding factor (LEF)/T-cell factor (TCF) transcription factors [11,12] to promote transcription of important downstream target genes, many of which have been implicated in cancer [13-15]. Aberrant activation caused by β-catenin or APC mutations leads to the constitutive activation of Wnt canonical pathway in human colorectal cancers [16-19].

The Wnt pathway is aberrantly activated in numerous cancers [20-22], including lung cancer [23,24]. Both Wnt-1 and Wnt-2 are up-regulated in non-small cell lung cancer (NSCLC) [25,26], whereas Wnt-7a is down-regulated in most lung cancer cell lines and tumor tissues [27]. Co-expression of both Wnt-7a and Fzd9 inhibits cell growth of NCSLC cell lines [27]. Moreover, DVL has been shown to be over-expressed in 75% of micro-dissected NSCLC tissues [28]. Approximately 85% of all sporadic and hereditary colorectal tumors show loss of APC function, resulting in stabilization of β-catenin [29,30]. Mutations of the tumor suppressor gene APC or β-catenin are rare in lung cancer [31,32] and the Wnt pathway may be activated upstream of β-catenin [32,33]. Furthermore, both sFRP1 and WIF1 genes are reportedly silenced in lung cancer tissues [34-36]. Taken together, these studies indicate the important roles of the Wnt pathway in lung carcinogenesis.

Knowledge regarding the regulation of specific Wnts and their corresponding receptors in lung cancer is lacking. It is not known in great detail which receptors are selectively expressed or the roles they play in the pathogenesis of lung cancer. We recently found that Wnt-2 was upregulated in NSCLC [25]. Therefore, we sought to build on this finding by investigating specific Wnt/Frizzled interactions in human cancer cell lines and in lung cancer tissue samples. We also examined whether a dnhWnt-2 construct reduces tumor growth in cancer cell lines and in a xenograft mouse model.

## Methods

### Cell lines and tissues

Human lung cancer cell lines A549 and A427 were obtained from American Type Culture Collections (ATCC) (Manassas, VA) and cultured in RPMI 1640 medium. Human kidney epithelial cell line 293 and human kidney transfected epithelial cell line (293T) were obtained from ATCC and cultured in Dulbecco’s modified Eagle’s medium (DMEM). All cell cultures were supplemented with 10% fetal bovine serum, penicillin (100 IU/ml), and streptomycin (100 μg/ml) and incubated in a humid incubator with 5% CO_2_ at 37°C.

Fresh lung tumor tissues and adjacent normal lung tissues from patients who underwent surgical resection for lung cancers were collected and snap-frozen in liquid nitrogen in the operating room. Tissue samples were kept at −170°C in a liquid nitrogen freezer before use. The study was approved by the Committee of Human Research at the University of California and informed consent was obtained from all patients.

### Semi-quantitative RT-PCR and quantitative RT-PCR

Total RNA from mouse xenografts, fresh lung cancer and paired adjacent normal tissue was extracted with TRIzol LS (Invitrogen, Carlsbad, CA). Total RNA from the various cell lines was isolated using Qiagen’s RNeasy extraction method (Valencia, CA).

For semi-quantitative analysis, reverse transcription-PCR was performed with 1 μg total RNA in a GeneAmp PCR system 9700 (Applied Biosystems, Foster City, CA) using SuperScript II One-step RT-PCR with Platinum Taq (Invitrogen, Carlsbad, CA) for 25 cycles, according to the manufacturer's instructions. Primers were obtained from Operon Biotechnologies (Alameda, CA). Primer sequences for the human Wnt-2 cDNA were 5′-GGATGCCAGAGCCCTGATGAATCTT-3′ (Forward) and 5′-GCCAGCCAGCATGTCCTGAGAGTA-3′ (reverse). Primers for the human Frizzled-8 cDNA were 5′ GGACTACAACCGCACCGACCT-3′ (forward), and 5′ ACCACAGGCCGATCCAGAAGAC-3′ (reverse). Primer sequences for human DVL-3, c-Myc, Cyclin D1 and Survivin were obtained from Operon Biotechnologies. The housekeeping gene glyceraldehyde-3-phosphate dehydrogenase (GAPDH) (5′ ATGGGGAAGGTGAAGGTCGG-3′ forward; and 5′-GACGGTGCCATGGAATTTGC-3′, reverse) was amplified as an internal control [18,37]. The ratio of band intensity of Wnt-2 and Frizzled-8 between fresh lung cancer and paired adjacent normal tissues was measured using Image J software (NIH, Bethesda, MD, USA).

For quantitative RT-PCR, first-strand cDNA was synthesized from total RNA by iScript cDNA synthesis (Bio-Rad, Hercules, CA) according to the manufacturer‘s instructions. Taqman RT-PCR analysis was performed on cDNA in a 384-well plate using Prism 7900HT Real-Time PCR System (Applied Biosystems, Foster City, CA). Primers and hybridization probes for Wnt-2 and Frizzled-8 (inventoried, chosen from the online catalog) were purchased from Applied Biosystems (Foster City, CA). The expression of each gene was assayed in triplicate and normalized to GAPDH.

### Plasmid DNA constructs

The human Wnt-2 expression construct was kindly provided by J. Kitajewski (Columbia University). The dominant-negative Wnt-2 construct was generated by PCR amplification of the full-length human Wnt-2 cDNA using primers flanking the N-terminal domain from residues 1–278. The amplified cDNA fragment was then inserted into the pEGFP-N1vector (BD Biosciences Clontech, Palo Alto, CA) upstream of the GFP epitope to generate the dnhWnt-2 construct. The rat frizzled-1 (rFzd1), rFzd2; mouse frizzled-3 (mFzd3), mFzd4, mFzd5, mFzd7, mFrizzled-8 and mFzd9 mammalian expression constructs were kindly provided by R. Nusse (Stanford University). The mFzd10 expression construct was kindly provided by E. Morrisey (University of Pennsylvania).

### Selection for stable clones

Stable cell lines were generated by transfection of the expression vectors (pGFP-N1-dnhWnt-2) and control vector (pGFP-N1) into A549 and A427 cell lines using Lipofectamine 2000 (Invitrogen, Carlsbad, CA) according to the manufacturer‘s instructions. Transfected cells were selected by culturing in complete medium supplemented with Geneticin at 400 μg/mL (Invitrogen) for approximately 1 month. The stable transfectants were isolated and expanded for further analysis.

### TOPflash assay

Luciferase assays for reporters were carried out using the Dual-Luciferase Reporter Assay System (Promega, Madison, WI) as reported previously [28]. Briefly, 293, 293T, A549 and A427 cell lines were plated in 96-well plates with fresh media without antibiotics 24 hr before transfection. Lipofectamine 2000 (Invitrogen, Carlsbad, CA) was used to mediate co-transfection of pTOPflash (0.2 μg) or pFOPflash (0.2 μg) vectors (kindly provided by H. Clevers, Netherlands Institute). The cell lines were co-transfected with or without the following expression constructs: Fzd, Wnt-2, dnhWnt-2 and empty vectors pcDNA3.1 (Invitrogen) or pEGFP-N1 (each at 0.2 μg; 0.6 μg DNA in total), as indicated. The Renilla luciferase reporter vector pRL-TK (0.02 μg) (Promega, Madison, WI) was simultaneously transfected as the control for transfection efficiency. TCF-mediated transcriptional activity was determined by the ratio of pTOPflash/pFOPflash luciferase activity, each normalized to the luciferase activities of the pRL-TK reporter. Cells were harvested 48 hr after transfection. The experiments were done in triplicate.

### Western blot analysis

Whole cell lysates of cell lines were extracted with CytoBuster Protein Extraction Reagent (Novagen, Madison, WI). Cytosolic proteins were prepared as previously described [38]. The proteins were separated on 4–15% gradient SDS–polyacrylamide gels and transferred to Immobilon-P membranes (Millipore, Bellerica, MA). The proteins were first bound with the following primary antibodies: β-catenin (Transduction Laboratories, Lexington, KY, USA) and β-actin (Sigma Chemical, St. Louis, MO). Antigen-antibody complexes were detected by using an ECL blotting analysis system (GE Healthcare Bio-Sciences*,* Piscataway*,* NJ). The ratio of band intensity of β-catenin to β-actin was measured using Image J software (NIH, Bethesda, MD, USA).

### Cell proliferation and colony formation assays

Cell proliferation was determined using the CellTiter 96 AQueous One Solution Cell Proliferation Assay (Promega, Madison, WI). Briefly, A549 cells were plated in a 6-well plate 24 hr before transfection. Transient transfection was carried out using 4 μg of the dnhWnt-2 construct or the pEGFP-N1 empty vector. Twenty-four hours after transfection, cells were seeded in a 96-well plate at a density of 5×10^2^ cells per well and cultured for another 24 hr period before the CellTiter 96 Aqueous One solution was added. The assay was repeated daily for 4 consecutive days. Cell viability was measured at absorbance 490 nm. Each experiment was done in triplicate and repeated at least three times. Colony formation was analyzed in stably transfected A549 and A427 cell lines. Cells (5×10^2^) were plated in 6-well cell-culture dishes and incubated in complete medium containing Geneticin (400 μg/mL) for a minimum of 14 days. The colonies were then stained with 0.1% crystal violet, and colonies were counted. Results were shown as the mean number of colonies formed with the presence of dnhWnt-2 or the empty vector control. Colony assays were performed a minimum of three times each.

### Tumor xenografts

All *in vivo* experiments were performed in accordance with UCSF institutional guidelines (Institutional Animal Care and Use Committee approval number: AN085516-01). Six week-old female nude mice, strain athymic Nu/Nu (Taconic, Hudson, NY) received subcutaneous injections of 5×10^6^ cells in 100 μl of RPMI 1640, together with 25 μl of Matrigel basement membrane matrix (Becton Dickinson, Bedford, MA). Mice were inoculated subcutaneously into the right flank with A549 stable clones expressing the dnhWnt-2 vector and into the left flank with A549 cells stably expressing the vector control. Tumors were measured twice weekly at their greatest length and width for approximately 6 weeks. Tumor volume was calculated according to *x*^2^*y*/2, where *x* <*y*, x = width and y = length, and was reported as the mean and standard deviation (SD) of five independent measurements (n = 5 mice each). After 43 days, tumors were resected and weighed. Total RNA was extracted from tumor tissues for RT-PCR analysis. Immunostaining against Ki67 was done on formalin-fixed, paraffin-embedded tumor specimens resected from day 43 xenograft mice to access the level of cell proliferation. Briefly, antigen retrieval was achieved in citrate buffer, and then blocked, followed by incubation with rabbit monoclonal Ki67 antibody (Thermo Fisher Scientific Fremont, CA). Sections were then incubated with secondary goat anti-rabbit antibody (Vector Laboratories, INC. Burlingame, CA) and counterstained with Hematoxylin. Ki67 proliferation was determined by the percentage of cells with positive nuclear staining. Cell nuclei (2,500) were counted on representative sections for each tumor type.

### Statistical analysis

Statistical analysis was performed using GraphPad Prism 6.0 for Windows. The values shown represent mean ± S.D. (error bars) of triplicate independent experiments. The difference between groups was determined by Student‘s *t*-tests and a *p* value ≤0.05 was considered statistically significant.

## Results

### Wnt-2 activation of frizzled receptors

**Figure 1:**
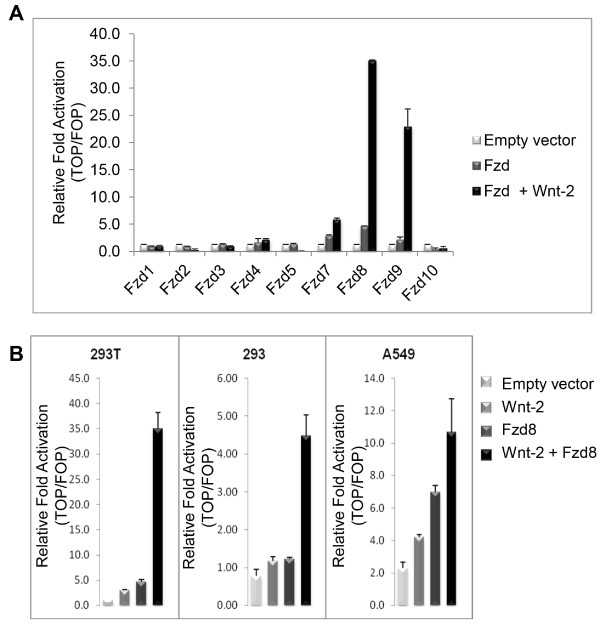
**Wnt-2 activation of Frizzled receptors. (A)** TCF transcriptional activity was measured in 293T cells transfected with the indicated Frizzled expression vectors in the presence or absence of Wnt-2 cDNA and vector control. Cells were co-transfected with pTOPflash or pFOPflash and internal control plasmid pRL-TK. Experiments were performed in triplicate and the level of expression was shown as relative fold activation (TOP/FOP) (mean ± standard deviation). **(B)** Activation of Frizzled-8 by Wnt-2 was measured in 293T, 293 and NSCLC cell line A549. TCF activity was determined in the cells transfected with Frizzled-8, Wnt-2, both Frizzled-8 and Wnt-2, or vector control. Experiments were performed in triplicate.

Wnt-2 is overexpressed in multiple cancers [23], but the specificity of the Wnt-2 interaction with its receptor(s) remains largely unknown. We therefore investigated Wnt-2 specificity by analyzing the abilities of several Frizzled receptors to induce T cell factor (TCF)-dependent transcription in the presence of Wnt-2. When Wnt-2 was co-expressed with each of the Frizzled receptors in 293T cells, TCF activity of Frizzled-8 increased by at least 25 fold over that of vector alone (Figure 1A). In addition, TCF activity of Fzd9 increased by ~15-fold over that of vector control alone, affirming previously reported data [39]. Frizzled-7 showed a 4-fold increase in TCF-activity compared to vector control and about a 2-fold increase due to the presence of Wnt-2. None of the other Frizzled expression vectors (Frz 3,4,5, etc.) showed increased activation after Wnt-2 co-expression (Figure 1A). We further analyzed this activation in normal epithelial 293 cells and NSCLC cell line A549. Wnt-2 activation of Frizzled-8 increased 5-fold in these cell lines compared to that of vector control (*p*<0.01) (Figure 1B). The empty vector control in A549 showed some activity, which is probably due to the intrinsic Wnt signaling in this cancer cell line. The results demonstrate for the first time that there is an interaction between Wnt-2 and Frizzled-8 in cancer cells.

### Up-regulation of Wnt-2 and frizzled-8 in lung cancer tissues

**Figure 2:**
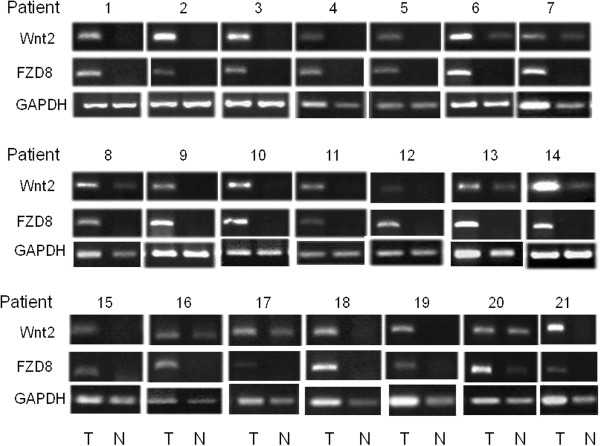
**Up-regulation of Frizzled-8 and Wnt-2 in lung cancer.** Semi-quantitative RT-PCR analysis of 50 freshly resected tumor samples with their corresponding matched, normal lung controls. Total RNA was reverse transcribed and amplified with primers specific for Wnt-2 or Frizzled-8. The data shown are representative tumor pairs (tumor (T) and normal (N)). Semi-quantitative RT-PCR products were resolved on a 1.5% agarose gel. Experiments were performed in triplicate.

The lung cancer tissues analyzed comprised 36 pairs of adenocarcinomas, 10 pairs of squamous cell carcinomas and 4 pairs of large cell carcinomas. Semi-quantitative RT-PCR analysis showed that Wnt-2 was up-regulated by 70% and human Frizzled-8 was up-regulated by 42% in the 50 lung tumor samples compared to their matched normal tissue controls. Furthermore, among the 21 lung tumor samples that had Frizzled-8 up-regulation, 91% showed up-regulation of Wnt-2 (*p*<0.05) (Figure 2).

### Inhibition of Wnt-2 signaling by dnhWnt-2

**Figure 3:**
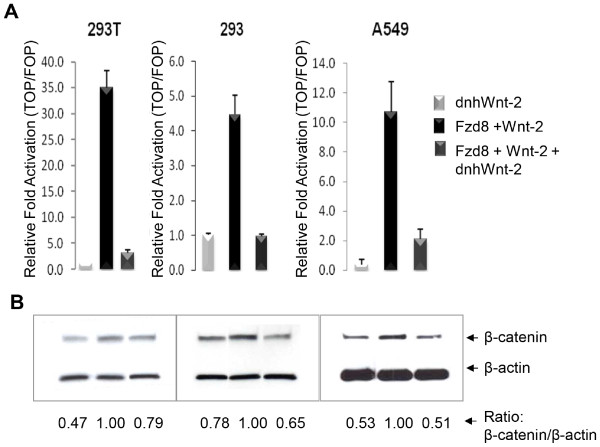
**Dominant negative human Wnt-2 inhibits Frizzled-8 activation. (A)** Inhibition of TCF transcriptional activity by dnhWnt-2 construct was measured in 293T, 293 and A549 cells. In the present or absence of dnhWnt-2 construct, the cells were co-transfected with both Frizzled-8 and Wnt-2 expression vectors, either pTOPflash or pFOPflash, and internal control plasmid pRL-TK. Experiments were performed in triplicate. **(B)** Stabilization of β-catenin by dnhWnt-2 expression in the indicated cell lines was determined by measuring cytosolic β-catenin protein levels in the cells.

We next sought to inhibit the effects of Wnt-2 activation of Frizzled-8 by designing a novel dnhWnt-2 construct. The human Wnt-2 gene was truncated at amino acid position 278, resulting in an 82 residue carboxyl terminal deletion generating the dnhWnt-2 construct. Co-expression of the dnhWnt-2 construct together with Wnt-2 and Frizzled-8 expression vectors in 293T and 293 cells strongly reduced TCF-dependent transcriptional activity, as determined by the TOPflash assay (Figure 3A). The 25-fold level of activation of Frizzled-8 by Wnt-2 observed in 293T cells was reduced to near vector control levels. Similarly, activation of Frizzled-8 by Wnt-2 in the 293 cell line was reduced. We further analyzed this activation in NSCLC cell line A549, and observed a decrease of TCF-dependent transcriptional activity by dnhWnt-2. The dnhWnt-2 alone inhibited the intrinsic Wnt (most likely Wnt-2) signaling and resulted in the low background of TCF activity in A549 cell line (Figure 3A). To determine if the dnhWnt-2 construct also affected β-catenin stabilization, we analyzed cytosolic β-catenin protein levels (Figure 3B). In all cell lines, β-catenin protein levels were elevated when cDNA of Frizzled-8 and Wnt-2 were co-expressed. However, dnhWnt-2 construct reduced cytosolic β-catenin protein levels to near background levels, even when Frizzled-8 and Wnt-2 were co-expressed.

### Effects of the dnhWnt-2 inhibitor in cancer cell lines

**Figure 4:**
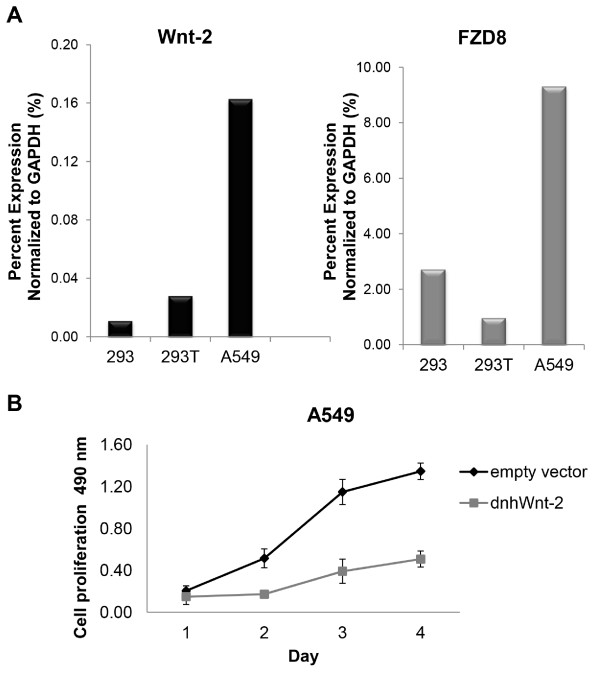
**Endogenous expression levels of Wnt-2/Frizzled-8 and effects of dnhWnt-2 expression in normal epithelial cells and NSCLC cells. (A)** Endogenous expression levels of Wnt-2 and Frizzled-8 were determined by quantitative RT-PCR analysis in 293, 293T and A549 cells. **(B)** Cell proliferation assays were performed in A549 cells transfected with dnhWnt-2 or vector control plasmids.

**Figure 5:**
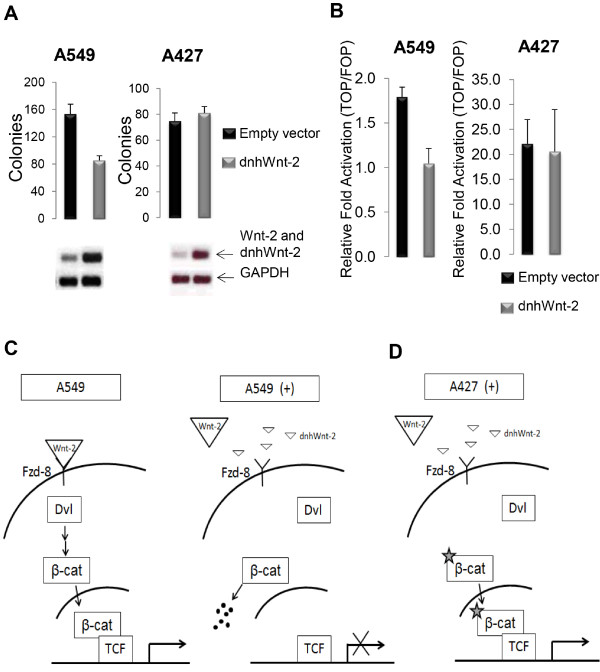
**Effects of dnhWnt-2 expression in NSCLC cell lines. (A)** Colony formation was determined in NSCLC cell lines A549 and A427. Expression of dnhWnt-2 construct and endogenous Wnt-2 was detected using semi-quantitative RT-PCR in these cell lines. **(B)** TCF transcriptional activity was measured in A549 and A427 cells in the present of dnhWnt-2 or vector control. Triplicate measurements were made and the level of expression was shown as relative fold activation (TOP/FOP) (mean ± standard deviation). **(C)** Models of Wnt-2 signaling regulated in NSCLC cell line A549. The Wnt canonical pathway is activated when endogenous Wnt-2 ligand (large triangle) binds to Frizzled-8 receptor (Fzd-8), which recruits the intracellular protein disheveled (Dvl) to plasma membrane. Activation of Wnt canonical pathway prevents the phosphorylation of β-catenin, resulting in the stabilization and translocation of β-catenin in the nucleus, where it activates target genes through binding to TCF transcription factors. In the presence (+) of dnhWnt-2 construct (small triangle), endogenous Wnt-2 is prevented from binding to Frizzled-8 receptor. The activation of Wnt canonical pathway is inhibited, resulting in the degradation of β-catenin and blockage of TCF transcriptional activity. **(D)** A model demonstrates Wnt-2 signaling regulation in NSCLC cell line A427, which harbors a β-catenin mutant (star). In A427 cells, the dnhWnt-2 construct competes the binding of Frizzled-8 receptor with endogenous Wnt-2 and inhibits the activation of Wnt canonical pathway. Instead of being phosphorylated and degraded, the β-catenin mutant is constitutively expressed and activates downstream Wnt signaling regardless of the presence of Wnt-2- ligand.

Since the dnhWnt-2 construct inhibited Wnt-2 signaling mediated by the Frizzled-8 receptor, we further investigated whether the dnhWnt-2 construct could inhibit cancer cell growth. Quantitative real-time RT-PCR confirmed that Wnt-2 and Frizzled-8 were endogenously overexpressed in NSCLC cell line A549 (Figure 4A) compared to normal epithelial 293 and 293T cells. A cell proliferation assay measured over a consecutive 4-day period in A549 cells showed that dnhWnt-2 mutant inhibited cell growth (Figure 4B). Wnt-2 was expressed in NSCLC cell lines A549 and A427, which were stably transfected with the dnhWnt-2 expression vector or the vector control vector. When dnhWnt-2 was expressed, the colony formation was reduced by 52% in the A549 cell line and was not affected in the A427 cell line (Figure 5A, top). PCR primers, which are specific to the sequence presented on both Wnt-2 and the dnhWnt-2 construct, were used for semi-quantitative RT-PCR analysis, and the expression of dnhWnt-2 and the endogenous Wnt-2 in A549 and A427 cells was confirmed (Figure 5A, bottom). TCF-mediated transcription was performed on the stable cell lines (Figure 5B). A549 cells expressing the dnhWnt-2 gene showed a 36% decrease (p<0.02) in activity compared to vector control cells. Based on our results, we have generated two hypothetical models. Model of Wnt-2 signaling in A549 cells (Figure 5C) shows that Wnt-2 binds to the Frizzled-8 receptor and activates Wnt-2 signaling in A549 cells (Figure 5C, left panel). The model also shows that dnhWnt-2 construct completes the binding with Wnt-2, resulting in the degradation of downstream β-catenin and the inhibition of TCF activity in A549 cells (Figure 5C, right panel). A model of Wnt-2 signaling in A427 cells (Figure 5D) shows that β-catenin mutant constitutively activates downstream Wnt signaling regardless of the presence of Wnt-2 ligand.

### Xenograft mouse model

**Figure 6:**
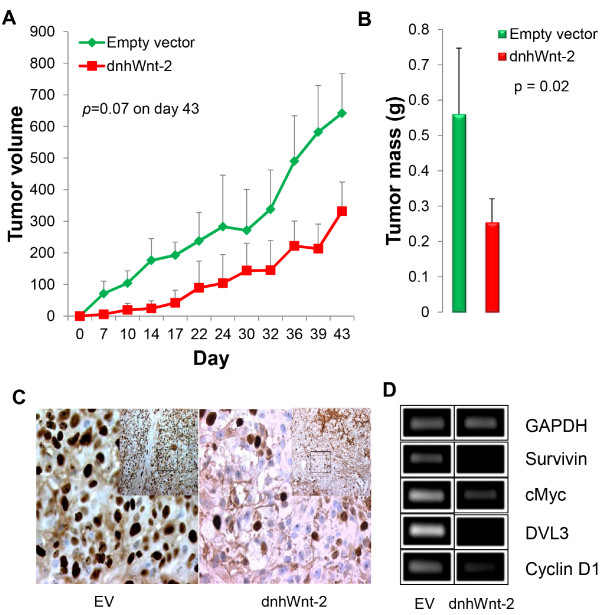
**Tumor xenograft. (A)** Tumor formation was monitored twice-weekly over a 43-day period in five groups of mice that received subcutaneous injections of A549 cells, which express dnhWnt-2 or vector control expression constructs (mean ± standard deviation, p = 0.007). **(B)** Tumor mass was measured on day 43 (mean ± standard deviation, p = 0.02). **(C)** Immunohistochemical analysis of cell proliferation by Ki-67 staining was performed in tumor sections expressing vector control (left panel) or the dnhWnt-2 (right panel) at 40× magnification. Inset is at 10× magnification. **(D)** qRT-PCR analysis of Wnt downstream target genes Survivin, c-Myc, DVL3, and Cyclin D1 in A549 xenograft tumors with either dnhWnt-2 or vector control. Expression of GAPDH was used as a control transcript.

A xenograft mouse model was generated with A549 cells stably expressing the dnhWnt-2 construct and vector control plasmid. The cells were transplanted into female athymic Nu/Nu mice and tumor formation was monitored twice per week. Tumor size and mass decreased significantly in the dnhWnt-2 tumors compared to tumor controls (n = 5) after 43 days of growth (*p*<0.05)(Figure 6A and 6B). Immunohistochemistry staining on tumor sections with Ki67 demonstrated cell proliferation at ~80% in control tumors compared to ~28% in dnhWnt-2 tumors (>2000 cell counts) (Figure 6C). Further analysis of the expression of Wnt downstream target genes in the dnhWnt-2 tumors (Figure 6D) showed that the expression of Survivin, c-Myc, Dvl-3 and Cyclin-D1 genes was down-regulated in dnhWnt-2 tumors compared to control tumors.

## Discussion

Wnt signaling is dysregulated in various tumors [21,22] and Wnt-2 has been suggested to play an oncogenic role in cancer [25,40]. Inhibition of Wnt signaling using different approaches has shown antitumor activity [22]. For instance, we previously reported that inhibition of Wnt-2 signaling using siRNA induces programmed cell death in NSCLC cells [25]. In the current study, we demonstrated for the first time that Wnt-2 signaling is activated through the Frizzled-8 receptor in NSCLC cells, and that a novel dnhWnt-2 construct reduces tumor growth in NSCLC cells and in a xenograft mouse model.

More recently, activation of Wnt signaling has been implicated in the metastasis of human cancer. In lung adenocarcinoma, activation of Wnt signaling has been shown to be a determinant of metastasis to brain and bone [41]. Moreover, enrichment of the Wnt-2 gene in circulating tumor cells was identified using RNA sequencing [40]. The association of Wnt-2 up-regulation with the formation of non-adherent tumors further suggests that Wnt-2 regulates metastasis of adherent tumors [40]. Our results suggest that therapeutic strategies targeting Wnt-2 signaling may prevent the development of metastasis and have potential impact on cancer mortality.

A dominant negative Wnt-8 construct has been shown to inhibit axis duplication induced by Wnt in the Xenopus model [42]. In our study, the dnhWnt-2 construct was designed by deleting an 82 amino acid truncation in the carboxyl-terminal of the human Wnt-2 gene. In our model, we demonstrated that dnhWnt-2 construct competes for the binding to the receptor(s) with Wnt-2, resulting in the degradation of cytoslolic β-catenin and the inhibition of TCF transcription in A549 cells (Figure 5C). In addition, our data indicate that the presence of dnhWnt-2 construct decreased cell proliferation and colony formation of A549 cells *in vitro*. We further analyzed the effect of dnhWnt-2 construct in lung cancer cell line A427, which harbors a mutation in the β-catenin gene and constitutively activates the β-catenin mutant (Figure 5D) [33]. As expected, dnhWnt-2 construct had a minimal effect on Wnt-2 signaling and colony formation in A427 cells. Although Wnt-2 is also expressed in A427 cells, its canonical signaling is probably more dependent on the β-catenin mutation and less dependent on the upstream signaling by Wnt ligands [43].

Although the frizzled family of receptors are known to function as key components of the Wnt signaling pathway [44], specific interactions of Wnt-2 with its receptor(s) have not been determined in lung cancer. In this study, we investigated the activation of Wnt-2 signaling through different Frizzled receptors. Our results show that both Frizzled-8 and Frizzled-9 were activated when Wnt-2 signaling was present in 293T cells. Overexpression of Frizzled-8 has been observed in lung cancer tissues and cell lines [45], and inhibition of Frizzled-8 expression using shRNA has been shown to reduce the proliferation of tumor cells *in vitro* and in a xenograft mouse model [45]. Frizzled-8 has been suggested to regulate Wnt signaling in lung cancer and can serve as a putative therapeutic target for the disease [45]. Frizzled-9 has also been shown to play a role in Wnt signaling. Rat Frizzled-9 receptor is activated by Wnt-2 and triggers the Wnt canonical pathway in 293T cells [39], which is consistent with our observation. Frizzled-9 is also activated in Wnt-7a signaling and functions as a tumor suppressor in lung cancer [27,46]. Whether the activation of Frizzled-9 receptor in Wnt-2 signaling is to promote or suppress the development of lung cancer is unknown. In addition to its role in oncogenesis, Frizzled-9 mediates the activation of Wnt-7a signaling in several developmental processes in normal tissue [47-49]. The function of Frizzled-9 in Wnt signaling is complex and its role in cancer development is not clear. In addition, Wnt3a was shown to signal through multiple Frizzled receptors in 293T cells [50], and Frizzled-5 appears to be the most active receptor for Wnt3a. In human cancer, Wnt3a appears to function both as oncogene and tumor suppressor gene in different cancer cell lines [51,52]. Further studies are needed to investigate the role of Wnt3a in lung cancer.

Inhibition of Wnt signaling has been shown to reduce tumor growth *in vitro* and in mouse models using a variety of approaches [25,47,53-58]. For instance, small molecules have been used to inhibit Wnt secretion or the transportation of β-catenin from the nucleus [47,55], and siRNA has been used to inhibit Wnt-2 signaling and induce apoptosis in NSCLC cells [25]. Fusion of Frizzled-8 CRD to human Fc can function as a soluble receptor *in vivo* and has been shown to inhibit tumor growth in xenograft models [59]. This antitumor activity mediated by Frizzled-8 CRD could partially result from the inhibition of Wnt-2 signaling. In this study, we used the dnhWnt-2 construct as a novel approach against lung cancer. Our results clearly show that the dnhWnt-2 construct reduces tumor growth in NSCLC cells and in a xenograft mouse model. Together, our findings, and those from other studies [25,59] strongly suggest that the further development of dnhWnt-2 construct will be useful in treating lung cancer.

## Conclusions

Our study demonstrates a strong correlation between the expression of Frizzled-8 and Wnt-2 in lung tumor samples. A robust TCF-dependent transcriptional activation in cell lines was observed when both Wnt-2 and Frizzled-8 are overexpressed. A novel dnhWnt-2 construct was designed and used to inhibit TCF-mediated transcription and colony formation when expressed in NSCLC cell line A549. Moreover, the dnhWnt-2 construct reduced tumor formation and the transcription of downstream target genes in a xenograft mouse model. Inhibition of Wnt-2 signaling with dnhWnt-2 construct may provide a new therapeutic avenue for targeting the Wnt pathway in lung cancer.

## Abbreviations

Fzd: Frizzled; NSCLC: Non-small-cell lung cancer; dnhWnt-2: Dominant negative Wnt-2; CRD: Cysteine-rich Wnt binding domain; DVL: Dishevelled; TCF: T-cell factor.

## Competing interests

The authors have no declared conflicts of interest.

## Authors’ contributions

Conceived and designed the experiments: DB LY. Performed the experiments: DB KK ZX. Analyzed the data: DB KK MH ZX LY YY DJ. Wrote the paper: DB YY LY. All authors read and approved the manuscript.

## Pre-publication history

The pre-publication history for this paper can be accessed here:

http://www.biomedcentral.com/1471-2407/13/316/prepub
